# RETRACTED: Bahmad et al. Histopathologic Findings Associated with Miller–Dieker Syndrome: An Autopsy Report. *Diseases* 2022, *10*, 95

**DOI:** 10.3390/diseases12010002

**Published:** 2023-12-21

**Authors:** Hisham F. Bahmad, Lauren Ramesar, Cecilia Nosti, Gameli Anthonio, Carole Brathwaite, Cristina Vincentelli, Amilcar A. Castellano-Sánchez, Robert Poppiti

**Affiliations:** 1 Department of Pathology and Laboratory Medicine, Mount Sinai Medical Center, Miami Beach, FL 33140, USA; cristina.vincentelli@msmc.com (C.V.); castelam@fiu.edu (A.A.C.-S.); robert.poppiti@msmc.com (R.P.); 2 Department of Translational Medicine, Herbert Wertheim College of Medicine, Florida International University, Miami, FL 33199, USA; lrame002@med.fiu.edu (L.R.); cnost001@med.fiu.edu (C.N.); ganth003@med.fiu.edu (G.A.); 3 Department of Pathology, Nicklaus Children’s Hospital, Miami, FL 33155, USA; carole.brathwaite@nicklaushealth.org

The Journal retracts the article, Histopathologic Findings Associated with Miller–Dieker Syndrome: An Autopsy Report [1]. 

Following publication, the authors contacted the editorial office and raised concerns regarding a lack of permission to publish personally identifying content within this article [1]. 

Adhering to our complaints procedure, an investigation was conducted by the Editorial Office and the Editorial Board that confirmed that the process for permission was not followed by the authors per MDPI’s Research Involving Human Subjects policy (https://www.mdpi.com/ethics#_bookmark9). Given that this article contains potentially identifiable and sensitive patient information (images and detailed medical history), the Journal, the Editor-in-Chief and the authors have taken the decision to retract and remove the article from MDPI’s website and relevant indexing databases in order to protect the identity of the patient and their family. In this situation, the article’s metadata (title and authorship information) and the retraction notice will be permanently retained. This decision is in line with MDPI’s retraction policy (https://www.mdpi.com/ethics#_bookmark30) and the Committee on Publication Ethics (COPE) guidelines (https://publicationethics.org/retraction-guidelines).

This retraction was approved by the Editor-in-Chief of the journal *Diseases*. 

The authors agreed to this retraction. 
